# Hypothalamic Alterations in Neurodegenerative Diseases and Their Relation to Abnormal Energy Metabolism

**DOI:** 10.3389/fnmol.2018.00002

**Published:** 2018-01-19

**Authors:** Pauline Vercruysse, Didier Vieau, David Blum, Åsa Petersén, Luc Dupuis

**Affiliations:** ^1^UMR-S 1118, Faculté de Médecine, Institut National de la Santé et de la Recherche Médicale (INSERM), Strasbourg, France; ^2^UMR-S1118, Université de Strasbourg, Strasbourg, France; ^3^Department of Neurology, Ulm University, Ulm, Germany; ^4^UMR-S 1172—JPArc, Centre Hospitalier Régional Universitaire de Lille (CHRU de Lille), Alzheimer and Tauopathies, Lille, France; ^5^Translational Neuroendocrine Research Unit (TNU), Lund University, Lund, Sweden

**Keywords:** hypothalamus, neurodegeneration, amyotrophic lateral sclerosis, Huntington’s disease, Alzheimer’s disease, fronto-temporal dementia, weight loss

## Abstract

Neurodegenerative diseases (NDDs) are disorders characterized by progressive deterioration of brain structure and function. Selective neuronal populations are affected leading to symptoms which are prominently motor in amyotrophic lateral sclerosis (ALS) or Huntington’s disease (HD), or cognitive in Alzheimer’s disease (AD) and fronto-temporal dementia (FTD). Besides the common existence of neuronal loss, NDDs are also associated with metabolic changes such as weight gain, weight loss, loss of fat mass, as well as with altered feeding behavior. Importantly, preclinical research as well as clinical studies have demonstrated that altered energy homeostasis influences disease progression in ALS, AD and HD, suggesting that identification of the pathways leading to perturbed energy balance might provide valuable therapeutic targets Signals from both the periphery and central inputs are integrated in the hypothalamus, a major hub for the control of energy balance. Recent research identified major hypothalamic changes in multiple NDDs. Here, we review these hypothalamic alterations and seek to identify commonalities and differences in hypothalamic involvement between the different NDDs. These hypothalamic defects could be key in the development of perturbations in energy homeostasis in NDDs and further understanding of the underlying mechanisms might open up new avenues to not only treat weight loss but also to ameliorate overall neurological symptoms.

## Introduction

Neurodegenerative diseases (NDDs) typically involve the degeneration of selective neuronal populations, leading to characteristic symptoms. The common NDDs include Alzheimer’s disease (AD), Huntington’s disease (HD), Parkinson’s disease (PD), frontotemporal dementia (FTD) and amyotrophic lateral sclerosis (ALS). Most NDDs exist in familial forms, associated with a family history and are genetically linked to disease-causing variants in known genes. For instance, familial AD can be caused by mutations in *APP*, *PSEN1* or *PSEN2*, while familial ALS can be associated with mutations in *SOD1*, *C9ORF72*, *TARDBP*, or *FUS*. Another example of familial NDD is HD, caused by an expansion of the trinucleotide CAG in the gene encoding huntingtin, leading to an expanded polyglutamine stretch in the huntingtin protein. The identification of gene variants associated with NDDs has allowed for the generation of transgenic mouse models commonly used to study NDDs.

Besides the classical symptoms, NDDs also feature other symptoms and signs, such as weight loss, endocrine perturbations, abnormal mood and altered behavior. Interestingly, many of these features could be caused by damage to the hypothalamus, a small brain structure that serves as an integration hub between the Central Nervous System (CNS) and its environment. Here, we will review the evidence of hypothalamic alterations in NDDs, with a specific focus on energy metabolism and body weight changes. We will first briefly introduce the anatomy of the hypothalamus and its most important functions. We will then detail the hypothalamic alterations in several NDDs, in particular AD, HD, FTD and ALS.

## Anatomy and Functions of the Hypothalamus Relevant to Neurodegenerative Diseases

**Figure 1:**
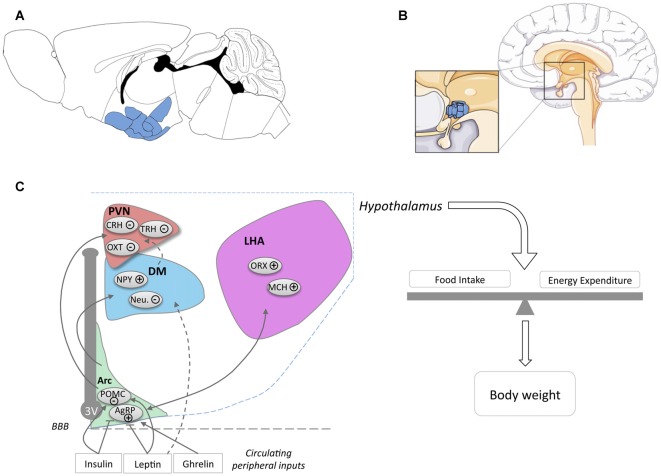
Location of the hypothalamus and its nuclei. Location of the hypothalamus in mouse **(A)** and human **(B)** brains. The panel **(C)** schematically presents the major hypothalamic nuclei and their involvement in energy homeostasis. For simplicity, cocaine and amphetamine regulated transcript (CART) expression was omitted from POMC neurons (noted POMC) and NPY expression was omitted from AgRP neurons (noted AgRP). Abbreviations: 3V, Third ventricle; ARC, Arcuate Nucleus; DM, Dorsomedial Nucleus; LHA, Lateral Hypothalamus Area; PVN, Paraventricular Nucleus; POMC, Pro-opiomelanocortin; AgRP, Agouti related peptide; NPY, neuropeptide Y; Neu., Neurotensin; ORX, Orexin; MCH, Melanin Concentrating Hormone; OXT, Oxytocin; TRH, Thyrotropin-releasing hormone; CRH, Corticotropin-releasing hormone; BBB, Blood Brain barrier.

The hypothalamus is a region of the CNS located on the ventral side of the brain symmetrically on both sides of the third ventricle (Figures 1A,B). The hypothalamus orchestrates signals from the CNS and the periphery and regulates basic body functions including reproduction, food intake, control of circadian rhythm and sleep-wake cycle. The hypothalamus is composed of several nuclei (Figure 1C), each of them with distinct functions.

An important function of the hypothalamus and the major focus in this review is the regulation of food intake and energy expenditure. The role of hypothalamic neurons in the regulation of energy balance is exquisitely complex and a detailed description goes far beyond the scope of the current review. Hence, we will briefly introduce the major nuclei and neuronal populations involved in the regulation of energy balance and would like to refer the reader to various reviews for further details (Berthoud, 2002; Berthoud and Münzberg, 2011; Yeo and Heisler, 2012; Morton et al., 2014; Schneeberger et al., 2014; Brown et al., 2015; Musiek and Holtzman, 2016; Caron and Richard, 2017; Timper and Brüning, 2017).

The hypothalamus plays a key role in the integration of multiple signals, either peripheral or central, in a coherent response, dependent on the metabolic status (Berthoud, 2002; Timper and Brüning, 2017). in situations of altered energy supply (e.g., fasting, post-prandial state), the hypothalamus orchestrates behavioral responses, such as adapted food intake, food-seeking behavior and optimizes energy use through behavioral adaptations and modified energy expenditure (Morton et al., 2014; Timper and Brüning, 2017). A myriad of peripheral cues exist that allow the hypothalamus to appropriately sense the metabolic situation. The most notable peripheral hormones are leptin, insulin and ghrelin. Leptin and insulin are key anorexigenic hormones, produced by adipocytes upon replenishment of adipose stores and by the endocrine pancreas, respectively. Ghrelin is produced by the gastroinstestinal tract and stimulates feeding. Multiple other peripheral hormones are involved in the regulation of metabolism and most of them signal to the hypothalamus. Moreover, hypothalamic neurons also senses alterations in nutrients such as glucose or fatty acids and are able to modify metabolic homeostasis.

The arcuate nucleus, or infundibular nucleus in humans, is located at the most ventral part of the hypothalamus, in the medial part bordering the third ventricle (Figures 1A,B) and is the major site of integration of peripheral cues. Indeed, leptin, insulin and ghrelin act directly on arcuate nucleus neurons, specifically on two major neuronal populations, so-called agouti related peptide (AgRP) and pro-opiomelanocortin (POMC) neurons (Figure 1C). AgRP neurons, co-expressing AgRP, neuropeptide Y (NPY) and GABA, are inhibited by leptin and insulin and activated by ghrelin. Their activation increases food intake and body weight (Schwartz et al., 2000; Morton et al., 2014). POMC neurons co-express POMC and cocaine and amphetamine regulated transcript (CART) and are activated by leptin and insulin. Among several neuropeptides, cleavage of POMC produces α-Melanocyte-stimulating hormone (α-MSH), that is mainly responsible for the anorexigenic effect triggered by the activation of POMC neurons. α-MSH is an endogenous agonist of melanocortin MC3 and MC4 receptors, while AgRP antagonizes this action. The integration of the melanocortin tone in projection neurons targeted by POMC and AgRP neurons leads to the regulation of energy balance through actions on food intake and energy expenditure.

The major sites of projection of POMC and AgRP neurons are the paraventricular nucleus (PVN), the lateral hypothalamus area (LHA), the dorsomedial hypothalamus (DM) and the ventro-medial hypothalamus (VMH). The PVN is located at the border of the third ventricle and is a major anorexigenic center, through the coordinated action of several neuropeptides including oxytocin (Blevins and Ho, 2013), vasopressin (Pei et al., 2014), corticotropin-releasing hormone (CRH) and thyrotropin-releasing hormone (TRH; Schwartz et al., 2000). The LHA, located on the most outer part of the hypothalamus (Schwartz et al., 2000; Berthoud and Münzberg, 2011) is considered to be a major site for hunger, through the action of two major neuronal types, melanin concentrating hormone (MCH) and Orexin (ORX)/hypocretin neurons. Both MCH and ORX neurons mediate increased food intake but are antagonistically regulating sleep wake cycles (Hillebrand et al., 2002; Gao and Horvath, 2007; Berthoud and Münzberg, 2011; Brown et al., 2015). DM and VMH are also targeted by ARC projections. The VMH is considered as a satiety center and densely expresses brain derived neurotrophic factor (BDNF) and together with its receptor TrkB are genetically associated with human obesity (Yeo and Heisler, 2012).

It is worth noting that energy balance is not only controlled by the hypothalamus alone but multiple extra-hypothalamic regions, including brainstem nuclei (e.g., nucleus tractus solitarius, raphe nuclei), or telencephalic structures (e.g., several cortical areas, ventral tegmental area…) are also involved in this complex function. In the last years, independent lines of research described alterations of the function and/or structure of hypothalamic nuclei in NDDs and began to decipher the functional consequences on disease progression. The next sections will detail these alterations in AD, HD, FTD and ALS.

## Hypothalamus and Alzheimer’s Disease

### Symptoms and Pathology of Alzheimer’s Disease

AD is a NDD usually starting during the sixth decade of life. The main AD symptoms are memory loss associated with impairments related to speech, personality, judgment, vision, association sensory-motor function (Castellani et al., 2010). Familial forms of AD represent less than 2%–3% of cases and are linked to mutations within amyloid precursor protein (*APP*), presenilin 1 (*PSEN1*) and presenilin 2 (*PSEN2*) genes (Ballard et al., 2011). Late-onset Alzheimer’s disease (LOAD) is a multifactorial and heterogeneous disorder associated with genetic and environmental risk factors. *APOE4* is the strongest genetic risk factor for LOAD (Holtzman et al., 2012).

From a neuropathological perspective, AD is defined by the presence of neurofibrillary tangles (NFT) made up of intraneuronal fibrillar aggregates of hyper- and abnormally phosphorylated tau proteins and the extracellular accumulation of Aβ peptides into amyloid plaques (Masters et al., 1985; Sergeant et al., 2008). Aβ is generated constantly through the sequential action of two proteases, beta and gamma secretases, which cleave APP (Querfurth and Laferla, 2010). The amount of Aβ in the cerebral tissue depends upon clearance mechanisms (reviewed in Wang et al., 2017). Failure of any of these clearance mechanisms in the brain, at least partly due to the disruption of the phagocytic properties of glial cells and parenchymal neuroinflammation, leads to an Aβ overload, toxic oligomers accumulation and formation of plaques (Heneka et al., 2015; Selkoe and Hardy, 2016). Regarding tau, its protein sequence contains more than 85 phosphorylated or phosphorylatable sites by more than 30 kinases including GSK3β, cdk5, JNK and AMPK (Sergeant et al., 2008). Hyperphosphorylation of tau leads to conformational changes that notably impair its ability to bind to microtubules. Free monomers of misfolded tau then start to accumulate, oligomerize and aggregate. Tau aggregates can deposit in NFTs that are observed early in life and increase during ageing (Braak et al., 2011). The spatiotemporal progression of NFT from the entorhinal cortex and the hippocampus to the isocortical areas has been shown correlated with cognitive deficits in the AD brain (Duyckaerts et al., 1997; Grober et al., 1999), supporting a pivotal role for tau pathology in AD-related memory impairments. Recent imaging studies particularly emphasized that while tau and Aβ topographies are spatially distinct and both correlate with disease progression, tau deposition more closely tracks dementia status and is a better predictor of cognitive performance than Aβ deposition (Brier et al., 2016).

### Hypothalamic Defects in AD

Several neuroimaging studies have observed hypothalamic atrophy in AD patients (Ishii and Iadecola, 2015). Callen et al. (2001) and Loskutova et al. (2010), demonstrated decreased hypothalamic volume of respectively 10% and 12% in their cohorts. Such hypothalamic atrophy appeared in early clinical stages of AD (Loskutova et al., 2010). In line with these findings, typical AD pathology such as amyloid plaques and NFTs, is regularly observed in the hypothalamus of AD patients (Ishii and Iadecola, 2015). Several hypothalamic nuclei have been described to exhibit amyloid plaques and NFTs (PVN, LHA, suprachiasmatic nucleus (SCN), tuberomammillary nucleus and the supraoptic nucleus; reviewed in Ishii and Iadecola, 2015).

At the cellular level, ORX neurons were reported to be decreased by 40%–50% in the LHA (Fronczek et al., 2012). Consistently, ORX levels were slightly decreased in the CSF of AD patients (Fronczek et al., 2012). SCN degeneration has also been reported in AD (Harper et al., 2008). This is consistent with sleep fragmentation which underline AD (Lim et al., 2014). More globally, dysfunctions of hypothalamic-pituitary-adrenal (HPA) axis, hypothalamic-pituitary-thyroid (HPT) axis and hypothalamic-pituitary-gonadal (HPG) axis have been consistently described in AD patients and may participate to the pathophysiological development of AD (Ishii and Iadecola, 2015). While dysregulation of all these systems may indirectly promote energy homeostasis changes, we will focus the following section on particular metabolic indexes namely body weight regulation and the relationship with glucose homeostasis.

### A Mutual Link between CNS Pathology and Peripheral Metabolism in AD?

Several modifiable risk factors have been identified for AD (Reitz et al., 2011; Livingston et al., 2017). Interestingly, among them, obesity, insulin resistance and diabetes significantly and independently increase AD risk (Profenno et al., 2010; Schrijvers et al., 2010). Indeed, abnormal energy metabolism is very frequently observed in AD patients. About 50%–60% of Alzheimer cases show abnormal eating behaviors (Ikeda et al., 2002) while 14%–80% of AD cases are of poor nutritional status (Droogsma et al., 2015). Further, weight loss is recognized as a clinical feature of AD in about 20%–45% of cases (Aziz et al., 2008b). In AD patients, cortical structures (e.g., parietal, posterior temporal, posterior cingulate-precuneal) show prominent hypometabolism. Whether and how this is related to weight loss remains unknown. Weight loss appears to be associated both with amyloid burden (Hsu et al., 2016) and disease progression (Cova et al., 2016). Importantly, weight loss is already present 10 years before the onset of the disease in presymptomatic gene carriers (Müller et al., 2017). Considering that plaques and tangles are found in the hypothalamus at stages III and IV corresponding to early-moderate AD and weight loss often occurs prior to cognitive impairments, factors other than tau and Aβ accumulation in the hypothalamus could contribute to such metabolic dysregulation. Furthermore, body mass index (BMI) decline in older age is associated with increased risk of developing AD as well as with a faster rate of disease progression (Aziz et al., 2008b).

The causes of weight loss and abnormal eating behavior during the clinical phase of AD remain unknown. While the metabolic changes could be related to the above-mentioned hypothalamic lesions, the signaling pathways involved are not known. Indirect impact from early locus coeruleus impairments could also contribute to this phenomenon (Rorabaugh et al., 2017 and references herein; Guimarães et al., 2012 for review). Some studies support the involvement of leptin signaling in the changes in energy homeostasis in AD (reviewed in Ishii and Iadecola, 2015). Aβ peptides have been found to be able to alter the response of arcuate nucleus NPY neurons to leptin (Ishii et al., 2014) but this remains controversial (reviewed in Ishii and Iadecola, 2015). Several studies also suggest that AD patients may have insulin resistance, and it is thus unlikely that body weight changes in AD patients are due to increased anorexigenic action of insulin (see below). Also, lesions of arcuate nucleus favors the development of AD-like lesions in experimental models (Dief et al., 2014; Špolcová et al., 2015). Weight loss could also be a resultant effect to defects in sensory integration or processing, in particular taste or olfaction.

Besides body weight loss, compelling evidence over the years supports a mutual relationship between glucose homeostasis changes and AD pathophysiology. As stated before, obesity, insulin resistance and diabetes are strong risk factors for AD. Experimental studies support the link between such metabolic disturbances and AD with impaired amyloid or tau pathologies being worsened following the development of obesity and insulin-resistance in transgenic AD models of amyloidogenesis or Tau pathology (Ho et al., 2004; Julien et al., 2010; Kohjima et al., 2010; Takeda et al., 2010; Leboucher et al., 2013; Moser and Pike, 2017). The mechanisms underlying these changes are not fully understood. Some studies argue that peripheral impairments are prone to favor brain insulin resistance and itself sufficient to promote the development of tau pathology and amyloidogenesis (Stanley et al., 2016) but this remains controversial (Leboucher et al., 2013). Pathological changes may also be, for instance, related to hypothermia (Gratuze and Planel, 2017; Gratuze et al., 2017; Tournissac et al., 2017).

Interestingly, the risk to develop diabetes has been suggested to be increased in AD patients (Janson et al., 2004), which support the notion of a mutual relationship between metabolic disturbances and AD lesions in the brain. Indeed, the concept that AD lesions can disrupt central mechanisms and are associated with peripheral impairments is emerging. The brain of AD patients has been shown to exhibit insulin resistance, exemplified by the increased cortical phosphorylation of serine inhibitory sites of IRS-1 which is correlated with cognitive score (Talbot et al., 2012). This observation is perfectly in line with the known ability of insulin signaling to promote plasticity and memory which may have relevance for changes in the cerebral cortex (Grillo et al., 2015) or the improvement of memory seen in humans after intranasal insulin administration. The potential involvement of brain insulin resistance in the development of glucose homeostasis impairment seen in AD patients is also supported by the known role of insulin signaling in energy metabolism regulation (Steculorum et al., 2014).

**Figure 2:**
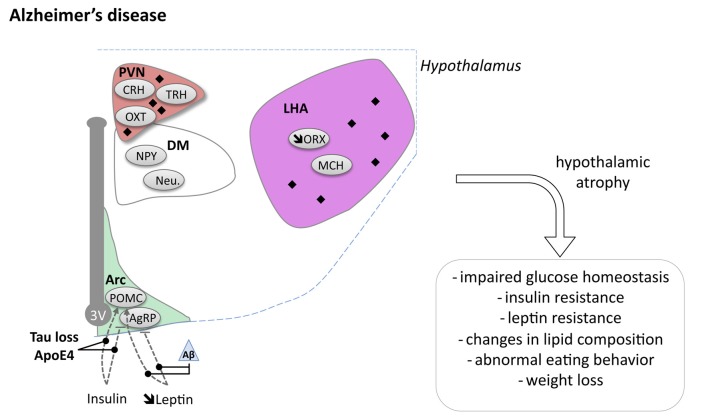
Hypothalamic involvement in Alzheimer’s disease (AD). The main neuropathological changes identified in the hypothalamus in postmortem tissue of AD patients are indicated. The pathological aggregates (either amyloid plaques or NFTs) are shown as black diamonds mostly in PVN and LHA. The dashed lines indicate a reduced effect. Loss of orexin neurons and a slight increase of MCH neurons have been reported. Abbreviations as in Figure 1, except: NFT, Neurofibrillary tangle; ROS, Reactive oxygen species; Aβ, Amyloid Beta; BACE1, beta secretase 1; APP, Amyloid precursor protein.

The origin of brain insulin resistance in AD patients seems to be related to both Aβ and tau pathologies. On the one hand, intracerebroventricular infusion of Aβ oligomers in mice is sufficient to trigger peripheral glucose intolerance by a hypothalamic based mechanism (Clarke et al., 2015). On the other hand, loss-of-function of tau, expected to occur concomitantly following hyperphosphorylation and aggregation of tau, impairs responsiveness to insulin and is associated with altered glucose homeostasis (Marciniak et al., 2017). This is in line with increased IRS-1 inhibition in the brain of patients with pure tauopathies (Yarchoan et al., 2014). Although the underlying mechanisms remain unclear, Aβ oligomers have been shown to promote insulin receptor internalization (Zhao et al., 2008) as well as activation of the JNK, PKR and TNFα pathways, in turn inhibiting IRS-1 function (Bomfim et al., 2012; Clarke et al., 2015; Lourenco et al., 2015). Moreover, tau has been suggested to promote IRS-1 function as well as PIP3 production, both favoring insulin signaling (Marciniak et al., 2017). Notably, recent data indicated that ApoE4 contributes to neuronal insulin resistance and could synergistically act together with lesions (Zhao et al., 2017). Altogether, these data emphasizes that glucose homeostasis impairments seen in AD patients are likely the result from lesions in the hypothalamus inducing a defective insulin signaling (Figure 2).

## Hypothalamus and Huntington’s Disease

### Symptoms and Pathology of Huntington’s Disease

HD is a monogenic neurodegenerative disorder that is inherited in an autosomal dominant pattern. It is caused by an expansion of the CAG triplet repeat in exon 1 of the Huntingtin (HTT) gene which leads to an expanded polyglutamine stretch in the ubiquitously expressed huntingtin protein (MacDonald et al., 1993). Short expansions (36–39 CAG repeats) increase the risk of developing the disease, while expansions larger than 40 CAG repeats lead to complete disease penetrance. The length of the CAG expansion negatively influences the age of onset which usually occurs in midlife (Ross et al., 2014). The disease affects about 10.6–13.7 individuals per 100,000 in Western populations, whereas the prevalence is as low as 1–7 per 10,00,000 in countries such as Japan and Taiwan (McColgan and Tabrizi, 2018). The clinical diagnosis is currently based on the presence of typical motor symptoms, including both involuntary movements (chorea, dystonia and athetosis) and disturbed voluntary movements (bradykinesia) in combination with a positive gene test (Ross et al., 2014). The survival rate is about 20–25 years after the disease diagnosis. Non-motor features of the disease are common and often present many years prior to motor dysfunction. The non-motor aspects include psychiatric symptoms, cognitive decline, sleep problems as well as metabolic alterations (Mochel and Haller, 2011; Paulsen et al., 2013; Ross et al., 2014; Epping et al., 2016).

The neuropathological classification of HD is based on a grading system from 0 to 4 based on neuronal loss and atrophy of the striatum of the basal ganglia (Vonsattel et al., 1985). One hallmark of the disease is the presence of mutant huntingtin containing intraneuronal inclusions (Difiglia et al., 1997). Whereas the motor symptoms in HD are associated with the progressive and pronounced dysfunction and cell death of medium sized projection neurons in the striatum, less is known about the neurobiological substrate underlying the non-motor features of the disease.

### Altered Metabolism in HD

Weight loss is a well-known clinical feature of HD, particularly in later stages (Djoussé et al., 2002; Mochel et al., 2007; Marder et al., 2009; Süssmuth et al., 2015). As weight loss has not been found to correlate with increased motor activity, it is possible that increased energy metabolism may be one explanation (Mochel et al., 2007; Süssmuth et al., 2015). In fact, patients with early disease stage and mild-moderate stage exhibit increased total energy expenditure (TEE) with higher basal resting energy expenditure (Goodman et al., 2008; Aziz et al., 2010a). Furthermore, presymptomatic HD mutations carriers consume more calories (Mochel et al., 2007), perhaps to compensate for increased metabolism. Interestingly, in contrast to what has been observed in control subjects, energy expenditure measured by indirect calorimetry has been found to further increase after insulin stimulation in HD patients (Aziz et al., 2010a). It is well known that insulin acts on the VMH to stimulate the sympathetic nervous system, a major regulator of the resting metabolic rate. In fact, the sympathetic nervous system is overactive in HD patients (Bellosta Diago et al., 2017). Hence, the increased impact of insulin stimulation on energy metabolism in HD patients suggests that sympathetic hyperactivity may be a contributing factor to altered systemic energy metabolism in HD. However, the underlying mechanisms of an overactive sympathetic nervous system in HD are not known.

Alterations in energy homeostasis may be important in the pathogenic process in HD. In fact, it has been repeatedly reported that HD patients with higher BMI at onset of symptoms have slower disease progression (Gilbert, 2009; Süssmuth et al., 2015). It is therefore possible that the underlying cellular and molecular mechanisms of altered metabolism in HD can provide novel targets for disease modifying therapeutic interventions (Duan et al., 2014).

A number of studies have investigated whether there are any altered levels of peripheral metabolic factors in HD. Although previous reports have shown increased levels of ghrelin, reduced levels of leptin and insulin resistance (Popovic et al., 2004; Lalić et al., 2008; Aziz et al., 2010b), a few recent studies have not been able to identify any altered metabolic markers in HD patients (Lazar et al., 2015; Nambron et al., 2016). There is currently no clear metabolic signature in the blood that can explain the altered energy metabolism in HD.

There are several animal models for HD that are genetically engineered to express varying lengths of the large *HTT* gene with different CAG repeats (Pouladi et al., 2013). However, direct comparisons between models are difficult as they are developed on diverse strain backgrounds using different promoters and gene constructs leading to varying expressing levels of the huntingtin protein of variable sizes and with different polyglutamine lengths. Nevertheless, most animal models of HD display metabolic alterations with either increases or decreases in body weight. This indicates that the presence of mutant huntingtin has an important impact on the regulation of energy metabolism. The commonly used R6/2 mouse model of HD that expresses around 150 CAG repeats in the exon 1 of human *HTT* gene (Mangiarini et al., 1996), reproduces the clinical findings of higher caloric intake, weight loss and hypermetabolism (Goodman et al., 2008; van der Burg et al., 2008). Knock-in models such as the ZQ175 mice develop weight loss, but further characterization when it comes to systemic energy homeostasis has not been published (Peng et al., 2016; Southwell et al., 2016). Mouse models that express the full length *HTT* gene with around 100 CAG repeats, the YAC128 and BACHD mice, display increased body weight and develop obesity with insulin and leptin resistance (Gray et al., 2008; Pouladi et al., 2010; Hult et al., 2011). Mutant huntingtin is expressed in all tissues and alterations in energy metabolism could therefore arise from both central and peripheral pathologies found in these animal models (van der Burg et al., 2009; Petersén and Gabery, 2012; Carroll et al., 2015). Hypothalamus is one affected area in HD that could cause metabolic imbalance. Interestingly, inactivation of mutant huntingtin in the hypothalamus prevented the development of the metabolic alterations in the BACHD mice, indicating that mutant huntingtin can alter energy metabolism by affecting hypothalamic pathways. Indeed, selective hypothalamic overexpression of mutant huntingtin of both short and longer fragment lengths in wild-type mice using recombinant adeno-associated viral (rAAV) vectors led to increased food intake and the development of severe metabolic alterations (Hult et al., 2011; Soylu-Kucharz et al., 2015). Furthermore, selective expression of mutant huntingtin fragments in the hypothalamus led to similar alterations in metabolism-regulating peripheral tissues such as brown adipose tissue as has previously been described in mouse models ubiquitously expressing mutant huntingtin (Weydt et al., 2006; Soylu-Kucharz et al., 2015). Hence, it is possible that mutant huntingtin affects important hypothalamic circuitries regulating energy metabolism in HD.

### Hypothalamic Defects in HD

**Figure 3:**
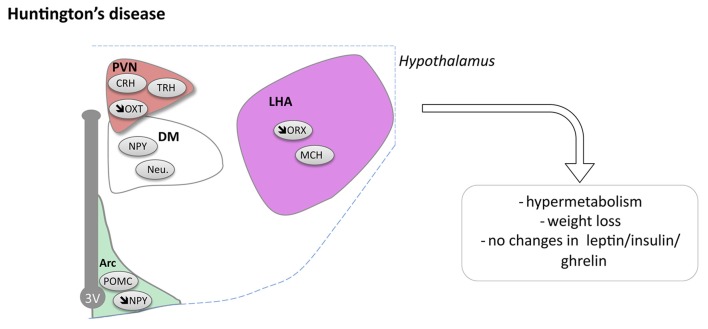
Hypothalamic involvement in Huntington’s disease (HD). The main neuropathological changes identified in the hypothalamus in postmortem tissue of HD patients and mouse models are indicated. Loss of orexin, oxytocin, vasopressin and NPY neurons have been reported. Abbreviations as in Figure 1.

Pathological changes in the hypothalamus are both present in HD patients and in relevant animal models (Petersén and Gabery, 2012). Stereological analyses of the total number of neurons in postmortem hypothalamic tissues from HD patients have not revealed any significant cell loss compared to control cases, but there may still be effects on smaller cell populations or in selective nuclei (Gabery et al., 2010). In fact, analyses of neuronal numbers in the PVN showed 23% neuronal loss in HD cases compared to controls (Gabery et al., 2010). Further neuropathological analyses investigating different neuropeptide-expressing populations have revealed loss of somatostatin neurons in the lateral tuberal nucleus, orexin (hypocretin) neurons in the LHA, NPY neurons in the infundibular nucleus as well as of oxytocin and vasopressin neurons in the PVN compared to control cases (Kremer et al., 1990, 1991; Timmers et al., 1996; Petersén et al., 2005; Aziz et al., 2008a; Gabery et al., 2010, 2015; van Wamelen et al., 2014; Figure 3). The increase in total number of CART expressing neurons has been reported whereas the number of neurons expressing MCH in the hypothalamus appears to be spared (Aziz et al., 2008a). Taken together, the existing data suggest that some hypothalamic neurons may be selectively sensitive to the expression of mutant HTT.

Reduced levels of the proprotein-convertases that are important enzymes for the conversion of precursor molecules into bioactive neuropeptides have been reported in the PVN and the infundibular nucleus in HD, providing further support for hypothalamic dysfunction in HD (van Wamelen et al., 2013). In prodromal HD patients, imaging studies using voxel based morphometry and mathematical modeling techniques with magnetic resonance imaging (MRI) as well as positron emission tomography (PET) have shown that the hypothalamus may be affected already before motor onset and that the pathological changes include loss of dopamine D2 receptors as well as microglia activation (Kassubek et al., 2004; Politis et al., 2008; Soneson et al., 2010). In line with this, a recent neuropathological case study of an individual with prodromal HD showed that oxytocin and vasopressin loss in the hypothalamus was present even before striatal pathology (Gabery et al., 2015). Further studies are warranted to identify the extent, selectivity and importance of hypothalamic changes in clinical HD.

Animal models provide a useful tool to dissect out mechanistic links related to human disease. Several animal models of HD reproduce the clinical postmortem findings in the hypothalamus. Loss of oxytocin and vasopressin in the PVN as well as loss of orexin in the LHA have been found in several animal models including the R6/2 mouse and mice expressing mutant huntingtin selectively in the hypothalamus (Kotliarova et al., 2005; Petersén et al., 2005; van der Burg et al., 2008; Wood et al., 2008; Yamanaka et al., 2010; Hult et al., 2011). Reduced NPY and increased CART mRNA levels have been found in the hypothalamus of BACHD mice (Hult Lundh et al., 2013). Experimental studies using rAAV vectors to either express or inactivate mutant huntingtin in the mouse hypothalamus have indeed provided support for a causative link between hypothalamic dysfunction and non-motor phenotypes such as metabolic alterations and depressive-like behavior in HD (Hult et al., 2011; Hult Lundh et al., 2013). However, experiments to inactivate mutant huntingtin in specific nuclei or cell populations in the hypothalamus of the floxed BACHD mouse model bred with mice expressing cre-recombinase under the VMH specific promotor SF-1, under the PVN specific promotor Sim-1, or the leptin receptor promotor, did not show any beneficial effects on the psychiatric or metabolic phenotype (Lundh et al., 2012; Baldo et al., 2014; Soylu-Kucharz et al., 2016). Further studies investigating the effects of mutant huntingtin inactivation specifically in the LHA of BACHD mice or studies using other HD animal models with a more pronounced hypothalamic pathology such as R6/2 would therefore be interesting for shedding further light into causal links between hypothalamic expression of huntingtin and disease progression in HD.

## Hypothalamus and Fronto-Temporal Dementia

### Symptoms and Pathology of Fronto-temporal Dementia

FTD is a NDD with onset between 45 and 65 years of age, and progression to death after diagnosis of 2–5 years. FTD is characterized by progressive deficits in behavior, executive function and language. FTD presents as three variants, the major one being termed behavioral-variant FTD (bv-FTD) also called frontal-variant FTD (fvFTD). Bv-FTD patients show personality changes including stereotypic behaviors, disinhibition (for example interaction with strangers without respect of boundaries), and sometimes apathy (for example less interest toward family and friends (Neary et al., 2005; Ahmed et al., 2016c)). FTD shares common genetic causes and clinically overlaps with ALS (see later). Mutations in *TARDBP, TBK1* or *C9ORF72* can cause ALS, FTD and ALS/FTD (Lattante et al., 2015; Taylor et al., 2016). Mutations in *MAPT*, encoding Tau, or *PGRN*, encoding progranulin, cause typical FTD and have never been associated to ALS until now (Baker et al., 2006; van Es et al., 2017).

### Changes in Eating Behaviors in FTD

FTD patients show high prevalence of alterations in eating behaviors. Indeed, changes in the quality of preferred foods, but also in the quantity of ingested food are found in more than 60% of cases at onset, and from 80% to 100% of cases during the course of the disease (Ikeda et al., 2002; Piguet et al., 2011; Ahmed et al., 2014a). In a recent study measuring food intake at breakfast (Ahmed et al., 2016b), bv-FTD patients were found to eat twice as much as controls. FTD patients also develop new eating habits, such as eating every day at same time or new oral behaviors such as chewing or smoking. Bv-FTD patients usually display an increased preference for sweet taste, often qualified by caregivers as a sweet food-seeking behavior (Ahmed et al., 2016b). These altered eating habits consequently lead to weight alterations, including higher BMI (Ahmed et al., 2014a,b,c, 2015). However weight alterations can go both ways: while 30% of bv-FTD cases had gained more than 7.5 kg, Ikeda et al. (2002) observed that 9% of cases had lost more than 7.5 kg. Indeed, the increase in BMI is less than expected given the increased food intake, raising the possibility that bv-FTD patients develop increased energy expenditure as well (Ahmed et al., 2017). Increased energy expenditure, is a hallmark of ALS, and it is currently thought that the clinical, genetic and pathological continuum between ALS and FTD also extends to a metabolic continuum, with FTD patients tending to gain weight and ALS patients lose weight despite both diseases sharing similar alterations in food intake, insulin resistance and increased energy expenditure (Ahmed et al., 2016c).

### Hypothalamic Defects in bv-FTD

In a small cohort, it has been reported that bv-FTD patients exhibit atrophy of the hypothalamus that occurs mostly in the posterior part of the hypothalamus including the LHA and dorsomedial nuclei (Piguet et al., 2011). Two recent studies, further confirmed this initial observation (Ahmed et al., 2015; Bocchetta et al., 2015) that by-FTD patients exhibit a 15%–20% loss of hypothalamus volume. Thus, hypothalamic atrophy is an early feature of the disease, which appears within the 2 years after diagnosis. Bocchetta et al. (2015) further segmented the hypothalamic region in sub-regions, and observed preferential atrophy in the subunits containing PVN, DMN and LH/mammillary body. Interestingly, FTD patients with the highest eating disturbances also presented the most important atrophy of posterior part of hypothalamus (Piguet et al., 2011; Ahmed et al., 2015).

Two subtypes of bv-FTD are distinguished on neuropathological criteria, FTD-TAU with TAU positive inclusions in so-called Pick bodies, and FTD-TDP with TDP-43 positive inclusions. Piguet et al. (2011) found that FTD-TAU patients have more hypothalamic aggregates than FTD-TDP patients. In FTD-TAU, tau-immunoreactivity is observed in the overall hypothalamus, while in FTD-TDP only few inclusions were found in the posterior part of the hypothalamus (Piguet et al., 2011). Moreover FTD-TAU patients have more severe hypothalamic atrophy than FTD-TDP patients vs. controls. In the tau group, neuronal loss was observed, whereas in the TDP group, even if there is atrophy, no changes were reported in the number of neurons. Though there is a neuronal loss in FTD-TAU cases, this was not due to changes in appetite-controlling peptide neurons (NPY, CART and orexin neurons; Piguet et al., 2011). Peripheral hormone levels and hypothalamic neuropeptide levels were measured in blood of bv-FTD (Ahmed et al., 2015). No changes were observed between bv-FTD and controls for leptin, CCK, ghrelin, PYY and oxytocin. However levels of AgRP in fasting condition were three times higher in bv-FTD in comparison to controls and AgRP and leptin correlated with BMI.

**Figure 4:**
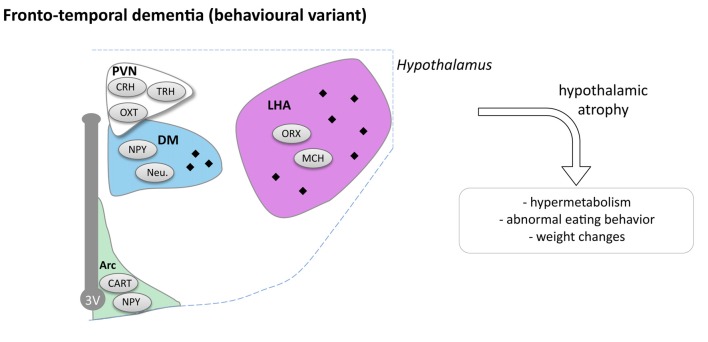
Hypothalamic involvement in fronto-temporal dementia (FTD; behavioral variant). The main neuropathological changes identified in the hypothalamus in postmortem tissue of bv-FTD patients are indicated. The pathological aggregates (TDP-43 or TAU aggregates) are shown as black diamonds mostly in LHA and DM. Abbreviations as in Figure 1.

In all, bv-FTD is specifically associated with hypothalamic defects that correlate with abnormal eating behaviors (Figure 4). It remains to be determined whether these hypothalamic changes are functionally involved in abnormal eating behaviors, or whether alterations in other brain regions, in particular frontal cortex, could constitute primary drivers of both altered behavior and hypothalamic defects. Experimental studies on animal models of bv-FTD are currently lacking to mechanistically link these independent observations.

## Hypothalamus and Amyotrophic Lateral Sclerosis

### Symptoms and Pathology of Amyotrophic Lateral Sclerosis

ALS is a NDD with onset between 50 and 65 years of age and an incidence of about three new cases per 100,000 individuals per year in Northern countries (Kiernan et al., 2011; Zarei et al., 2015; Couratier et al., 2016; van Es et al., 2017). ALS is fast progressing, with death of most patients within the first 3 years after onset. ALS is initially characterized by muscle twitching and/or cramping or speech or swallowing difficulties followed by spreading of muscle weakness and muscle atrophy, ultimately leading to death usually due to respiratory failure (Zarei et al., 2015). ALS symptoms are usually explained by the combined degeneration of Upper Motor Neurons (UMNs) and Lower Motor Neurons (LMNs) in the brain and spinal cord (Zarei et al., 2015).

### Metabolic Problems in ALS

In the last 20 years, it was shown that ALS patients are generally lean with normal or lower BMI. O’Reilly et al. (2013) found that a lower BMI from baseline is associated with a higher rate of developing ALS. Consistently, overweight (BMI from 25 to 29.9 kg/m^2^) and obese (BMI > 30.0 kg/m^2^) people have lower risk of ALS. A large European population study (Gallo et al., 2013) observed that underweight women (BMI < 18.5 kg/m^2^) have a higher risk of dying from ALS and increased BMI in men is associated with a decreased risk of ALS. Consistently, leptin levels were inversely related with ALS risk (Nagel et al., 2017). ALS patients at the presymptomatic stage without motor deficit, have a lower BMI, at least in the few years before the onset of motor symptoms (Huisman et al., 2015). However, a recent study by Peter et al. (2017) suggested that weight loss begins about 10 years before motor symptoms and is preceded by a period in which ALS patients are slightly overweight as compared with controls. In ALS, even with an appropriate caloric intake, patients lose weight (Nau et al., 1995; Kasarskis et al., 1996; Vaisman et al., 2009) and this weight reduction is as such not solely due to low nutritional intake. In all, weight loss can be considered as an early symptom that progresses over the disease course.

Several causes might underlie the energy imbalance in ALS. First, ALS patients consume more energy at rest when normalized by their mass composition. Approximately 50% (Bouteloup et al., 2009) to 60% of ALS patients (Desport et al., 2006) show increased energy expenditure (Desport et al., 2001; Bouteloup et al., 2009; Funalot et al., 2009; Vaisman et al., 2009). Increased energy expenditure has been demonstrated in patients with familial and sporadic ALS (Dupuis et al., 2011) throughout disease progression in a stable manner until proximity of death (Desport et al., 2006; Bouteloup et al., 2009). Second, about 50% of ALS patients (Kostic Dedic et al., 2012) display hyperlipidemia (Dupuis et al., 2008), and glucose intolerance with or without insulin resistance (Pradat et al., 2010; Dupuis et al., 2011). Third, the progression of motor symptoms to bulbar muscles is a major cause of dysphagia in ALS patients, thus reducing their capacity to eat (Kasarskis et al., 1996; Kühnlein et al., 2008; Vaisman et al., 2009). At the presymptomatic stage, ALS patients have a higher daily energy intake to compensate for increased energy expenditure, but that may augment the risk of hyperlipidemia and insulin resistance (Huisman et al., 2015). However, ALS patients seem to have normal food intake after disease onset (Ahmed et al., 2016a).

Mouse models of ALS also display typical metabolic problems. In two well characterized ALS models, *Sod1 (G86R)* and *SOD1 (G93A)* mice exhibit a lower body weight due to increased energy expenditure, in turn leading to the loss of body fat and decreased circulating leptin levels before the onset of motor symptoms (Dupuis et al., 2004). Similarly, mice overexpressing mutant TDP-43, *Tdp-43(A315T)* mice, as well as knock-in *Tdp-43(A315T)* mice display weight loss (Esmaeili et al., 2013; Stribl et al., 2014). In line with this, conditional *Tdp-43* Knock-Out mice (Chiang et al., 2010) show large decreases in body weight and body fat mass caused by a reduction in food intake and an increased fat oxidation. Interestingly, *Tdp-43(A315T)* overexpression (Stallings et al., 2013) led to weight gain before the onset of motor deficit, with an increase of fat mass in both white and brown adipose tissues. Interestingly, this symptom seems to be due to TDP-43 expression in peripheral target organs (like skeletal muscle, maybe fat) rather than in the CNS.

Weight loss in ALS was negatively correlated with disease severity using ALSFRS (Park et al., 2015) and disease progression (Kasarskis et al., 1996; Desport et al., 1999; Dupuis et al., 2011). Indeed, a correlation between BMI and disease progression has been well established. Higher BMI is correlated with a longer survival (Desport et al., 1999, 2000; Jawaid et al., 2010; Paganoni et al., 2011). In addition, hyperlipidemia appears to be correlated with survival (Dupuis et al., 2008) in a manner that seems dependent upon body weight loss (Paganoni et al., 2011; Kostic Dedic et al., 2012). In animal models, dietary rescue of weight loss was evaluated as a neuroprotective strategy. Increasing energy intake was sufficient to compensate for the increased energy expenditure, and to reduce the energy deficit of *Sod1 (G86R)* mice (Dupuis et al., 2004). More importantly high-energy diet reverted markers of muscle denervation, prevented loss of motor neurons in the spinal cord and increased the survival of 20% (Dupuis et al., 2004). In a phase 2 trial, adults with ALS receiving percutaneous enteral nutrition, gained three times more weight with high-carbohydrate diet hypercaloric (HC/HC) than control diet and showed slower decline of functional score and increased survival (Wills et al., 2014). However, this study involved only very few patients and included only patients with advanced disease. A larger clinical trial enrolling ALS patients earlier in their disease progression is needed to ascertain whether a nutritional therapy can be beneficial to patients.

### Hypothalamic Defects in ALS

Pathological TDP-43 inclusions were observed in the hypothalamus of about one third of ALS patients studied (Cykowski et al., 2014). The presence or absence and the density of inclusions were not correlated with disease duration in this case study of 30 ALS patients. Moreover, BMI of patients was similar whether or not they displayed inclusions in the hypothalamus. However, the presence of aggregates in the LHA was associated with a lower BMI. We recently performed a large MRI study comprising of 251 sporadic ALS cases, 19 symptomatic, 32 presymptomatic mutation carriers and 112 healthy controls (Gorges et al., 2017). A clear hypothalamic atrophy of about 22% was found in ALS patients as well as in presymptomatic gene carriers compared to controls. The atrophy was distributed through the anterior and posterior hypothalamus and not correlated with brain atrophy in ALS patients or clinical disease progression. However, the atrophy of the hypothalamus was associated with BMI, especially in familial cases of ALS and the atrophied anterior part of hypothalamus correlated with the age at onset (Gorges et al., 2017). Thus, the hypothalamus suffers from a region-specific degenerative process that is already present in the pre-symptomatic carrier, indicative of a pre-motor symptom process. Moreover, a smaller hypothalamic volume is linked to an earlier disease onset and the hypothalamus could thus be implicated not only with weight problems associated with ALS but also with the disease onset.

Recently, a *post hoc* analysis of clinical samples from patients treated with the anti-diabetic pioglitazone (Dupuis et al., 2012) provided more evidence of hypothalamic dysfunction in both ALS patients and mouse models (Vercruysse et al., 2016). Pioglitazone has well-documented peripheral effects and was shown to increase food intake through inhibition of hypothalamic MCH neurons (Diano et al., 2011). Indeed, pioglitazone robustly leads to a 3–5 kg weight gain in humans. While pioglitazone displayed all expected peripheral effects in ALS patients, it did not lead to weight gain in patients. Consistently, administration of pioglitazone to *Sod1 (G86R)* mice did not increase food intake in the same manner as compared to wild type mice (Vercruysse et al., 2016). This could be due to intrinsic defects in the hypothalamic MCH neurons as we observed multiple defects in POMC and AgRP neurons in these mice (Vercruysse et al., 2016).

**Figure 5:**
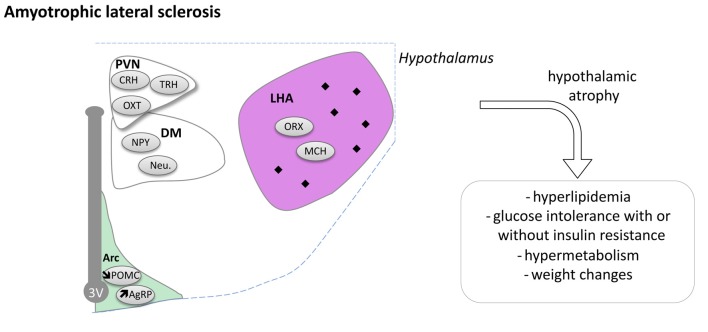
Hypothalamic involvement in Amyotrophic Lateral Sclerosis (ALS). The main neuropathological changes identified in the hypothalamus in postmortem tissue of ALS patients and mouse models are indicated. The pathological aggregates (TDP-43 inclusions) are shown as black diamonds mostly in LHA. Of note, the loss of POMC and increased AgRP expression were only shown in mouse models. Abbreviations as in Figure 1.

Taken together, a large body of evidence is now available indicating that the hypothalamus is compromised during ALS disease progression (Figure 5). How this relates to the spreading of the disease and whether these alterations constitute a relevant therapeutic target remain open questions that needs to be answered.

## Conclusion

Metabolic alterations, such as weight loss or insulin resistance, have been documented in most NDDs. In many cases, these alterations were causally associated with at least part of the neurological phenotype and shown to contribute to disease progression. Moreover, multiple epidemiological results also point to these defects as being premorbid to the actual neurological symptoms.

What could be the cause of metabolic imbalance during neurodegeneration? The hypothalamus is a key player in metabolic homeostasis and is damaged in multiple, non-overlapping ways in a number of NDDs. Indeed, hypothalamic changes have been directly observed in most NDDs (AD, PD, HD, bvFTD and ALS). In AD, HD, bvFTD and ALS, hypothalamic alterations have been observed either in presymptomatic gene carriers or in very early patients. Furthermore, pathological aggregates were found in the hypothalamus of most NDD patients and multiple neuropeptidergic populations are lost in the hypothalamus, potentially leading to the observed metabolic phenotype, but also contributing to other non-metabolic symptoms. Intriguingly, pathological aggregates and neuronal loss seem to target mostly the lateral hypothalamus.

Despite this large body of pathological and imaging evidence, very few studies to date seek to determine the functional role of hypothalamic alterations in both disease progression and dysfunction in energy metabolism. With the variety of conditional transgenic mice to model the different NDDs available, it will be possible in the near future to characterize whether specific hypothalamic neuronal populations are contributing to disease phenotype, in an extension of what has been initiated in the HD field. Moreover, the use of modern viral tracing tools, with optogenetics and pharmacogenetics, that revolutionized the field of obesity and diabetes, should be extended to the characterization of hypothalamic connectome in neurodegeneration. This is critical as a constellation of drugs has been developed in the field of diabetes, and some of them could be therapeutically useful to treat selected NDD.

In all, it is our opinion that the hypothalamus is a still underappreciated hub in the clinical picture of NDDs. Future preclinical research should investigate whether targeting hypothalamic pathways could be therapeutically useful in NDDs, both for slowing down weight loss and as a more general neuroprotective strategy.

## Author Contributions

All authors wrote the manuscript. PV drafted the figures.

## Conflict of Interest Statement

The authors declare that the research was conducted in the absence of any commercial or financial relationships that could be construed as a potential conflict of interest.
